# The CHC22 Clathrin-GLUT4 Transport Pathway Contributes to Skeletal Muscle Regeneration

**DOI:** 10.1371/journal.pone.0077787

**Published:** 2013-10-30

**Authors:** Sachiko Hoshino, Kazuho Sakamoto, Stéphane Vassilopoulos, Stéphane M. Camus, Christine A. Griffin, Christopher Esk, Jorge A. Torres, Norio Ohkoshi, Akiko Ishii, Akira Tamaoka, Birgit H. Funke, Raju Kucherlapati, Marta Margeta, Thomas A. Rando, Frances M. Brodsky

**Affiliations:** 1 The G.W. Hooper Foundation, Departments of Bioengineering and Therapeutic Sciences, Pharmaceutical Chemistry, and Microbiology and Immunology, University of California San Francisco, San Francisco, California, United States of America; 2 Department of Neurology, Faculty of Medicine, University of Tsukuba, Tsukuba, Ibaraki, Japan; 3 Department of Pharmacology, School of Medicine, Fukushima Medical University, Fukushima, Japan; 4 Department of Health, Faculty of Health Sciences, National University Corporation Tsukuba University of Technology, Tsukuba, Ibaraki, Japan; 5 Departments of Genetics and Medicine, Harvard Medical School, Boston, Massachusetts, United States of America; 6 Department of Pathology, University of California San Francisco, San Francisco, California, United States of America; 7 Department of Neurology and Neurological Sciences, Stanford University School of Medicine, Stanford, California, United States of America; University of Birmingham, United Kingdom

## Abstract

Mobilization of the GLUT4 glucose transporter from intracellular storage vesicles provides a mechanism for insulin-responsive glucose import into skeletal muscle. In humans, clathrin isoform CHC22 participates in formation of the GLUT4 storage compartment in skeletal muscle and fat. CHC22 function is limited to retrograde endosomal sorting and is restricted in its tissue expression and species distribution compared to the conserved CHC17 isoform that mediates endocytosis and several other membrane traffic pathways. Previously, we noted that CHC22 was expressed at elevated levels in regenerating rat muscle. Here we investigate whether the GLUT4 pathway in which CHC22 participates could play a role in muscle regeneration in humans and we test this possibility using CHC22-transgenic mice, which do not normally express CHC22. We observed that GLUT4 expression is elevated in parallel with that of CHC22 in regenerating skeletal muscle fibers from patients with inflammatory and other myopathies. Regenerating human myofibers displayed concurrent increases in expression of VAMP2, another regulator of GLUT4 transport. Regenerating fibers from wild-type mouse skeletal muscle injected with cardiotoxin also showed increased levels of GLUT4 and VAMP2. We previously demonstrated that transgenic mice expressing CHC22 in their muscle over-sequester GLUT4 and VAMP2 and have defective GLUT4 trafficking leading to diabetic symptoms. In this study, we find that muscle regeneration rates in CHC22 mice were delayed compared to wild-type mice, and myoblasts isolated from these mice did not proliferate in response to glucose. Additionally, CHC22-expressing mouse muscle displayed a fiber type switch from oxidative to glycolytic, similar to that observed in type 2 diabetic patients. These observations implicate the pathway for GLUT4 transport in regeneration of both human and mouse skeletal muscle, and demonstrate a role for this pathway in maintenance of muscle fiber type. Extrapolating these findings, CHC22 and GLUT4 can be considered markers of muscle regeneration in humans.

## Introduction

The recently-characterized isoform of clathrin in humans, known as CHC22, plays a specific role in sorting the GLUT4 glucose transporter to an insulin-responsive intracellular compartment in skeletal muscle and fat [1]. Insulin-stimulated release of GLUT4 from this GLUT4 storage compartment (GSC) to the plasma membrane enables glucose uptake by these tissues in which GLUT4 is preferentially expressed, constituting the major pathway of post-prandial glucose clearance from human blood [2]–[6]. Prior to definition of its specific function in GSC formation, our studies of CHC22 showed elevated levels in rat muscle undergoing regeneration after cardiotoxin injury [7]. Independently, other elements of the GLUT4 glucose uptake pathway have been implicated in rat muscle regeneration. Following cardiotoxin injury of rat muscle, GLUT4 expression is enhanced in regenerating fibers [8], as is expression of the vesicle-associated membrane protein-2 (VAMP2, also known as synaptobrevin), which mediates fusion of GLUT4-containing vesicles with the plasma membrane upon release from the GSC [9], [10]. VAMP2 expression is also enhanced in rat satellite cells [11], the muscle-associated cells that mediate regeneration of adult skeletal muscle [12]. These coincidental findings suggested that the GLUT4 glucose import pathway controlled by CHC22 might play a role in muscle regeneration. Here we address this hypothesis through analysis of regenerating human muscle tissue and muscle regeneration in transgenic mice expressing CHC22, two systems in which the CHC22-GLUT4 interaction can be more readily studied than rat muscle due to species restrictions of available antibody and genetic tools.

Skeletal muscle regeneration occurs continuously to repair muscle damage incurred during normal activity and is enhanced in response to disease or injury [12]. When regeneration cannot compensate for disease-induced deterioration, dystrophies and other chronic myopathies ensue. During adult regenerative myogenesis, satellite cells, which are normally quiescent, are stimulated by injury to proliferate, differentiate and fuse with one another or with existing myofibers to restore normal tissue architecture. Specialized membrane traffic plays a role in the development and maintenance of skeletal muscle function [13]. However, the membrane trafficking pathways involved in skeletal muscle regeneration are relatively uncharacterized.

Here, we address a role in skeletal muscle regeneration for CHC22, a second clathrin heavy chain isoform encoded on human chromosome 22. In contrast to the ubiquitous form of clathrin (CHC17 isoform), which is responsible for receptor-mediated endocytosis and sorting in the trans-Golgi network, CHC22 mediates a specific step in membrane traffic during retrograde endosomal sorting and does not operate in endocytosis [14]. CHC22 is present at low levels in many cell types, but its expression is highest in skeletal muscle, where retrograde endosomal sorting contributes to sequestration of GLUT4 in the GSC. In fact, CHC22 expression increases upon differentiation of myoblasts and adipocytes [1], [15] as they begin to express GLUT4 and form an insulin-responsive GSC, suggesting that CHC22 is a key player in the GLUT4 pathway. Notably, however, the murine gene encoding CHC22 clathrin evolved into a pseudogene, though it is present in other vertebrates [16]. Consequently, mice lack CHC22 and instead form their insulin-responsive GSC using transport pathways mediated by CHC17 [17]. Another membrane traffic protein absent from mice that operates in the same retrograde transport pathway, syntaxin 10 [18], is also required for human GSC formation [14]. When expressed as a transgene encoded by its human tissue-specific promoter in mouse muscle and fat, CHC22 causes aberrant localization of GLUT4 and VAMP2 and formation of an expanded GSC with impaired function [1]. Thus, CHC22 is needed for functional trafficking of both GLUT4 and VAMP2 to the GSC in human tissue, but its presence in mouse tissue disrupts trafficking pathways that normally proceed in its absence, conferring diabetic symptoms on the CHC22-mice [1].

Our early studies of CHC22 analyzed rat muscle by immunofluorescent labeling with an antibody against CHC22 and detected an increase in CHC22 immunostaining upon regeneration after cardiotoxin injection [7]. However, apart from microscopy evidence, we were not able to isolate CHC22 protein from rats, possibly due to weak cross-reactivity of antibodies recognizing human CHC22. To circumvent this problem and clarify a potential role for CHC22 and GLUT4 membrane traffic in muscle regeneration in the present study, we analyzed GSC pathway markers in human muscle sections from patients with four different myopathies including inflammatory myopathies, which are characterized by abundant regenerating myofibers [19]. Regenerating human myofibers showed elevated levels of CHC22, GLUT4 and VAMP2 compared to mature fibers and we observed elevated GLUT4 and VAMP2 in regenerating mouse myofibers. Furthermore, we found that the perturbation of GLUT4 and VAMP2 traffic in muscle of mice expressing CHC22 as a transgene was associated with impairment of muscle regeneration following cardiotoxin injection and with a change in muscle fiber type. Finally, we observed that cultured myoblasts from CHC22 trangenic mice could fuse into myotubes but did not proliferate in response to glucose, which stimulated wild-type myoblast proliferation. Together these observations identify a functional GLUT4 transport pathway as a component of skeletal muscle regeneration and myogenesis in both humans and mice.

## Materials and Methods

### Ethics Statement

Human muscle samples from patients with polymyositis, dermatomyositis, limb girdle muscular dystrophy, necrotizing myopathy and from control subjects were from the neuropathology archives of the University of Tsukuba and UCSF. For the samples from Tsukuba, the patient identity was coded to protect patient confidentiality. The study was approved by the Ethics Committee of University of Tsukuba Hospital and we confirm that written informed consent was obtained from patients for the use of their tissue in research. For the samples from UCSF, the study design was approved by the UCSF Committee on Human Research and the informed consent requirement was waived based on the non-invasive nature of the study and a minimal potential for harm to study participants. For animal experiments, all procedures were conducted in accordance with UCSF and National Institutes of Health guidelines, as approved by the UCSF Institutional Animal Care and Use Committee.

### Antibodies

Monoclonal and polyclonal antibodies against CHC22 have been described [1], [15]. Mouse anti-VAMP2 antibody was from Synaptic Systems (Goettingen, Germany). Rabbit polyclonal anti-GLUT4 was a gift from Jeffrey Pessin (Albert Einstein College of Medicine, Bronx) and mouse monoclonal anti-IRAP was a gift from Morris Birnbaum (University of Pennsylvania). Goat polyclonal anti-GLUT4 was from Santa Cruz (Santa Cruz, CA) and mouse monoclonal anti-GLUT4 was from Sigma (St. Louis, MO). Mouse monoclonal antibody against embryonic myosin heavy chain (F1.652) was from the Developmental Studies Hybridoma Bank, University of Iowa. Mouse monoclonal antibody against Pax7 (1G11) was from Sigma. Antibodies against Type I (clone NOQ7.5.4.D/MAB1628) and Type II (clone MY32) myosin heavy chains were from Millipore, MA and Abcam, Cambridge, UK respectively. Antibodies to CHC17 clathrin (monoclonal X22 against the heavy chain and polyclonal against the light chain subunits) were produced in the Brodsky laboratory and their characterization published previously [20], [21].

### Human Tissue and Quantification

To identify UCSF cases for the study, we performed a computerized search of the UCSF neuropathology case database using terms “dermatomyositis” and “necrotizing myopathy”, spanning the interval between 2007 and 2013. Three muscle biopsies were chosen for each disease category based on (1) absence of unusual pathologic or clinical features, (2) availability and quality of frozen muscle tissue, and (3) abundance of regenerating muscle fibers observed in hematoxylin and eosin-stained cryosections (Figure S1). The Tsukuba cases were selected in a similar fashion. The Tsukuba University neuropathology case database for 2000–2005 was searched manually for terms “polymyositis”, “dermatomyositis” and “limb girdle muscular dystrophy”, and muscle biopsies were chosen for each disease category. Muscle biopsy tissue of patients without final clinical or histological evidence of a myopathy served as normal controls.

**Figure 1:**
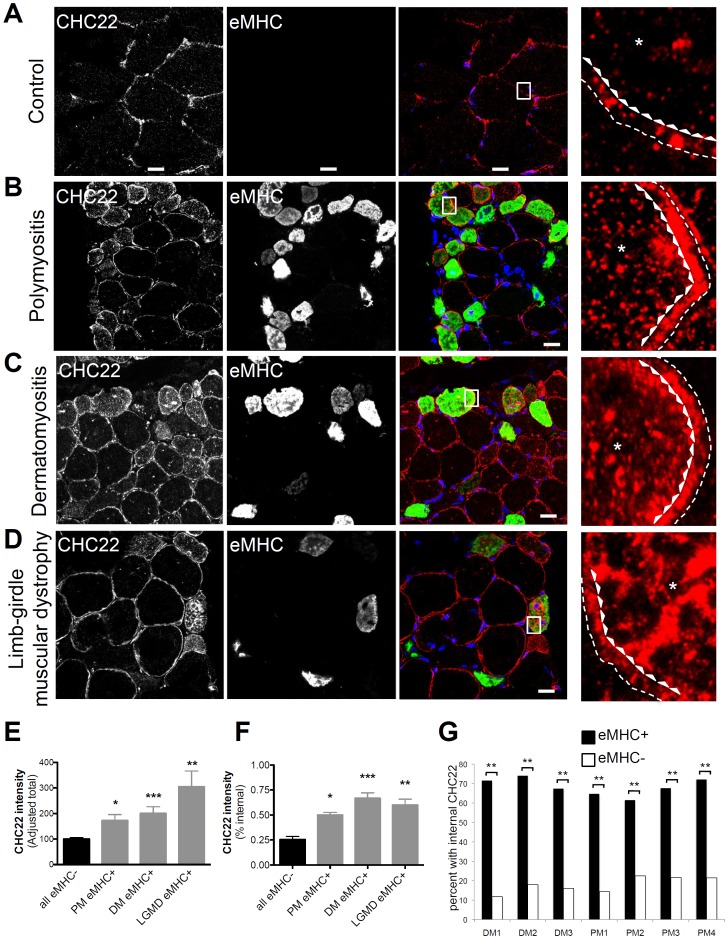
Increased internal CHC22 in regenerating muscle fibers in several human myopathies. A, B, C and D) Transverse sections of human skeletal muscle from a control individual or patients with polymyositis (PM), dermatomyositis (DM) or limb girdle muscular dystrophy (LGMD) were immunostained with polyclonal antibody against CHC22 (red) and a monoclonal antibody against embryonic myosin heavy chain (eMHC, green). Black and white images for each antibody are shown. In the merged color images nuclei were stained with DAPI (blue) and red-green overlap is shown in yellow (scale bars, 20 µm). The boxed regions in the merged images are magnified seven-fold at the far right and show CHC22 staining only. To illustrate the way in which internal fiber staining was quantified, the fiber boundaries drawn are shown in the magnified boxed regions with the thin dashed line representing the fiber border and the line of arrowheads representing the boundary for quantifying internal staining with the arrowheads pointing to the fiber interior. Only a segment of the fiber quantified is shown, but the asterisks in the internal region highlight the increased internal staining in the eMHC-positive fibers compared to the control. E) Quantification of total CHC22 fluorescence intensity (adjusted for patient comparison, see methods) in eMHC-negative (eMHC−) fibers (*n* = 40) from all patients and eMHC-positive (eMHC+) fibers from patients with PM (*n* = 17), DM (*n* = 14) or LGMD (*n* = 5) in the patients shown in A–D, where *n* is the number of fibers analyzed for each patient. All patients had statistically significant higher CHC22 fluorescence intensity in eMHC+ fibers (solid grey bars) compared to eMHC− fibers (solid black bar), as determined by one-way ANOVA (*p<0.05, **p<0.01, ***p<0.001). F) Quantification of internal CHC22 (internal pixel values/total pixel values with internal staining cut-off 2.5 microns beneath the fiber border, as illustrated in A–D in eMHC-negative (eMHC−) fibers (*n* = 19) from all patients and eMHC−positive (eMHC+) fibers from patients with PM (*n* = 8), DM (*n* = 4) or LGMD (*n* = 3) in the patients shown in A–D, where *n* is the number of fibers analyzed for each patient. Internal CHC22 fluorescence was significantly higher in eMHC+ fibers from patients with PM, DM and LGMD as determined by one-way ANOVA (*p<0.05, **p<0.01, ***p<0.001). G) The frequency of muscle fibers with internal CHC22 labeling was determined for fibers that were eMHC+ with central nuclei or eMHC− fibers in muscle sections from patients with PM (*n = *4) and DM (*n* = 3), immunostained as in A, where n is the number of patients analyzed. All patients analyzed had a statistically significant higher frequency of internal CHC22 in eMHC+ fibers (solid bars) than in eMHC− fibers (open bars) (**p<0.01), as determined by Fisher’s exact test.

**Figure 2:**
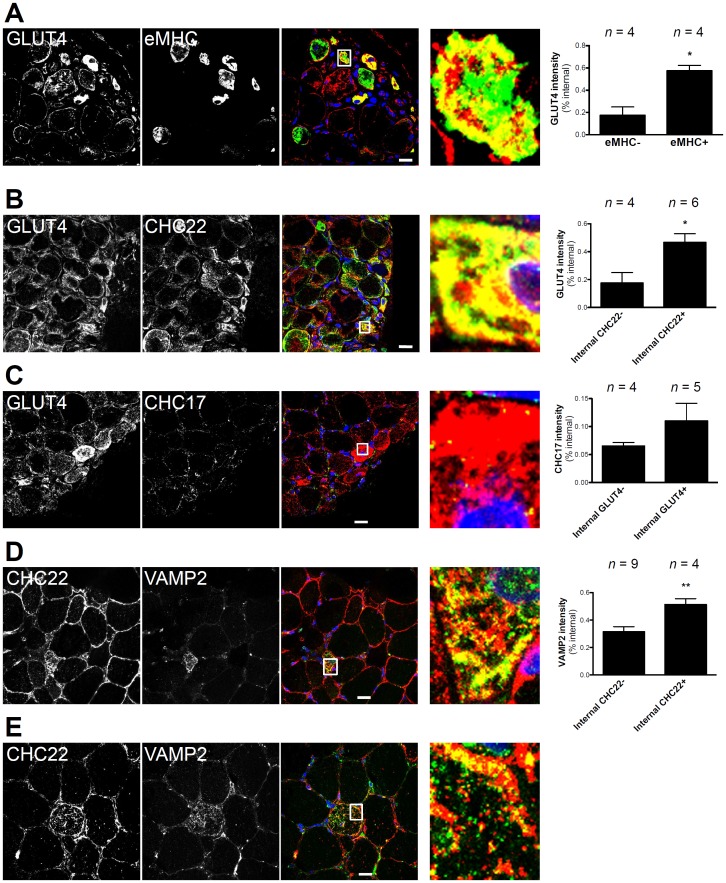
Co-localization of CHC22 and components of the GLUT4 trafficking pathway in regenerating human muscle. A, B and C) Transverse sections of skeletal muscle from a patient with PM were immunostained with a polyclonal antibody against GLUT4 (red) and monoclonal antibodies against A) embryonic myosin heavy chain (eMHC, green), B) CHC22 (green) or C) CHC17 (green). Regenerating myofibers were identified by centrally located nuclei stained with DAPI (blue in merge). Boxed regions in the merged images are magnified seven-fold at the right showing overlap of markers in yellow (scale bars, 20 µm). D and E) Transverse sections of skeletal muscle from a patient with PM were immunostained with a polyclonal antibody against CHC22 (red) and a monoclonal antibody against VAMP2 (green). Regenerating myofibers were identified by centrally located nuclei stained with DAPI (blue in merge). Boxed regions in the merged images are magnified seven-fold at the right showing intracellular co-localization (yellow) of CHC22 and VAMP2 in a regenerating fiber of D) small diameter or E) large diameter (scale bars, 20 µm). At the far right, the percent internal staining for the markers indicated on the y-axis was quantified as in Figure 1F, for fibers that were characterized according to internal staining for the marker indicated on the x-axis. *n* is the number of fibers analyzed in each staining combination shown at the left. Significant increased internal staining was determined by Student’s t-test (*p<0.05, **p<0.01).

In Figures 1 and 2, total fluorescence intensity per fiber was calculated using Image J as follows: Total = (integrated pixel values per fiber) – (area of fiber × mean background fluorescence). Adjusted total was determined to compare samples from different patients and was calculated by setting the average total fluorescence value of the eMHC− fibers for each patient to the arbitrary number of 100, then total fluorescence of the eMHC+ fibers in each patient was adjusted by the same factor used to set the eMHC− fibers to 100. For quantification of internal fluorescence intensity for each fiber, a line was drawn around the fiber boundary (dotted line in magnified panels of Figure 1) and a second line was drawn 2.5 microns from this in the direction of the interior of the fiber (line formed by arrowheads pointing to the interior in magnified panels of Figure 1). The integrated fluorescent signal inside the interior line was divided by the integrated fluorescence signal inside the exterior line to calculate the percent internal intensity. For the quantification in Figure 1G, five random fields from each patient section (*n* = 4 for PM and *n* = 3 for DM) were scored for the presence of eMHC and intense internal CHC22 staining (approximately 800–1300 total fibers per patient).

**Figure 3:**
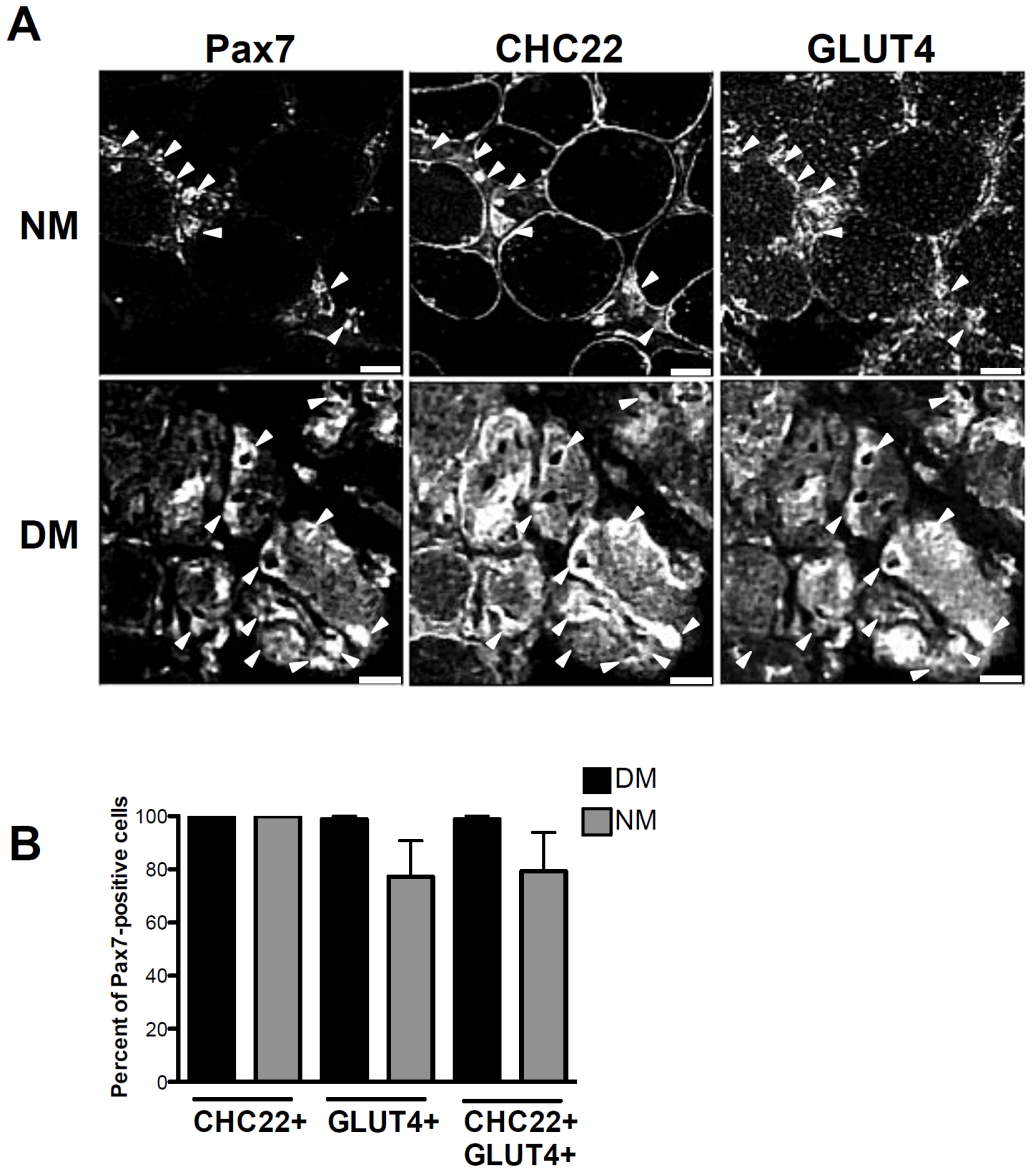
CHC22 and GLUT4 in satellite cells expressing Pax7. A) Transverse sections of human skeletal muscle from patients diagnosed with dermatomyositis (DM) or necrotizing myopathy (NM) were immunostained with a monoclonal antibody against Pax7, a polyclonal (rabbit) antibody against CHC22 and a polyclonal (goat) antibody against GLUT4. Arrowheads indicate Pax7+ CHC22+ GLUT4+ cells. (scale bars, 50 µm) B) The frequency of Pax7-positive cells with increased CHC22 and/or GLUT4 labeling was quantified for sections from DM (*n* = 3) and NM (*n* = 3) patient samples, immunostained as in A.

For the quantification in Figure 3, sections from patients diagnosed with dermatomyositis (*n* = 3) or necrotizing myopathy (*n* = 3) were analyzed. For each patient, 10 to 17 different fields were acquired and total, Pax7^+^, Pax7^+^CHC22^+^, Pax7^+^GLUT4^+^ and Pax7^+^CHC22^+^GLUT4^+^ cells were counted. Results were expressed as proportion of total or Pax7^+^ cells with approximately 500–1500 cells per patient scored.

### Cardiotoxin Injection

0.1 ml of 10 µM cardiotoxin (Sigma, St. Louis, MO) in 0.9% saline was injected directly into the right TA muscle and 0.1 ml of 0.9% saline was injected into left TA muscle with a 27-gauge needle under isoflurane anesthesia. The needle was inserted into TA muscle longitudinally from ankle to knee and was held in place for a few seconds, then slowly withdrawn along the long axis of the TA muscle with a little pressure to allow permeation of the cardiotoxin throughout the muscle. On day 1, 3, 5, 7, 14, 20, 28, and 56 after injection, six mice were sacrificed with an intraperitoneal injection of ketamine/medetomidine followed by cervical dislocation, and the cardiotoxin-injected TA muscles (right) and saline-injected contralateral TA muscles (left) were removed for analysis. The muscles from three mice per time point were frozen in isopentane cooled by liquid nitrogen for histological analysis. Histological analysis was performed quantitatively as described after standard hematoxylin and eosin (H & E) staining. The cross-sectional area and diameter of regenerating muscle fibers with central nuclei (500±150) in three sections from each mouse (three mice for each time point) were measured using Image J 1.38x, then, mean cross-sectional area or diameter was calculated for each time point. The muscles from the three additional mice per time point were frozen directly in liquid nitrogen for protein expression analysis and stored at −80°C. Mice used for these experiments were 8 weeks of age.

### Immunoblot Analysis

For lysates prepared from freshly isolated mouse skeletal muscle, tissue was homogenized with a polytron (3 times 5 s, speed 5) in lysis buffer (1 g/7 ml 50 mM Tris-HCl pH 7.5, 0.15 M NaCl, 1 mM EDTA, 1% NP40 and 1 protease inhibitor cocktail tablet (1/10 ml buffer, Roche)). Homogenate was centrifuged (10 min, 14,000×g) and the pellet discarded. Protein concentration of the lysate was determined by Bradford assay (Biorad). Lysates of human myoblast cultures were prepared as described [14]. Protein samples were separated by electrophoresis (10% acrylamide gel), then electrophoretically transferred to nitrocellulose (Millipore, Billerica, MA). The presence of CHC22 or other proteins in samples was assayed by immunoblotting, using a chemiluminescent reagent (Western Lightning Chemiluminescence Reagent Plus; PerkinElmer Life Sciences, Waltham, MA). Secondary antibodies were conjugated to horseradish peroxidase (Jackson ImmunoResearch Laboratories, West Grove, PA). Quantification was performed using Quantity One software (Biorad, Hercules, CA).

### Immunolabeling

Human skeletal muscle biopsy samples were embedded in Tissue-Tek OCT compound (Miles Inc., Elkhart, IN), frozen, and stored at −80°C (Tsukuba samples) or mounted using tragacanth gum, frozen in isopentane cooled by liquid nitrogen, and stored at −80°C (UCSF samples). Cryosections (5–10 µm thick) were collected on microscope slides, fixed for 10 min with 4% paraformaldehyde in PBS at room temperature and then washed twice (10 min each) with PBS at 4°C. After a 5-min permeabilization with 0.5% Triton X-100 in PBS at room temperature and a 30-min saturation with PBS supplemented with 0.1% Triton X-100, 0.5% bovine serum albumin, the sections were incubated overnight at 4°C with the primary antibodies in PBS supplemented with 0.1% Triton X-100, 0.5% bovine serum albumin, and 2% goat serum and washed three times (10 min each) at 4°C with PBS and 0.1% Triton X-100. The sections were then incubated with secondary antibodies for 30 min at room temperature. After three 10-min washes in PBS and 0.1% Triton X-100 at room temperature, the nuclei were stained with DAPI, and the samples were mounted with an anti-fading solution (DABCO). For double labeling, the two primary antibodies (from different species) and the two secondary antibodies were added at the same time. The secondary antibodies were labeled with either Alexa-488, Alexa-568 (both from Molecular Probes, Carlsbad, CA), or Cy3 (Jackson ImmunoResearch Laboratories, West Grove, PA). Images of muscle sections were acquired by confocal laser scanning microscopy (Nikon EZ-CiSi operating system and Leica DM1 6000 CS, SP5). Alexa-488, Alexa-568, or Cy3 fluorescence was sequentially excited and collected (400 Hz line by line) by using a 488-nm argon laser line for Alexa-488 and a 561-nm helium-neon laser line for Alexa-568 and Cy3 excitation. Fluorescence emission was collected from 493 to 555 nm for Alexa-488 and from 566 to 630 nm for Alexa-568 and Cy3. Part of the data for this study were acquired at the Nikon Imaging Center at the Mission Bay campus of UCSF.

### Transgenic Mice

Generation of the CHC22 transgenic mice is detailed elsewhere [1].

### Human Myotube Culture

**Figure 4:**
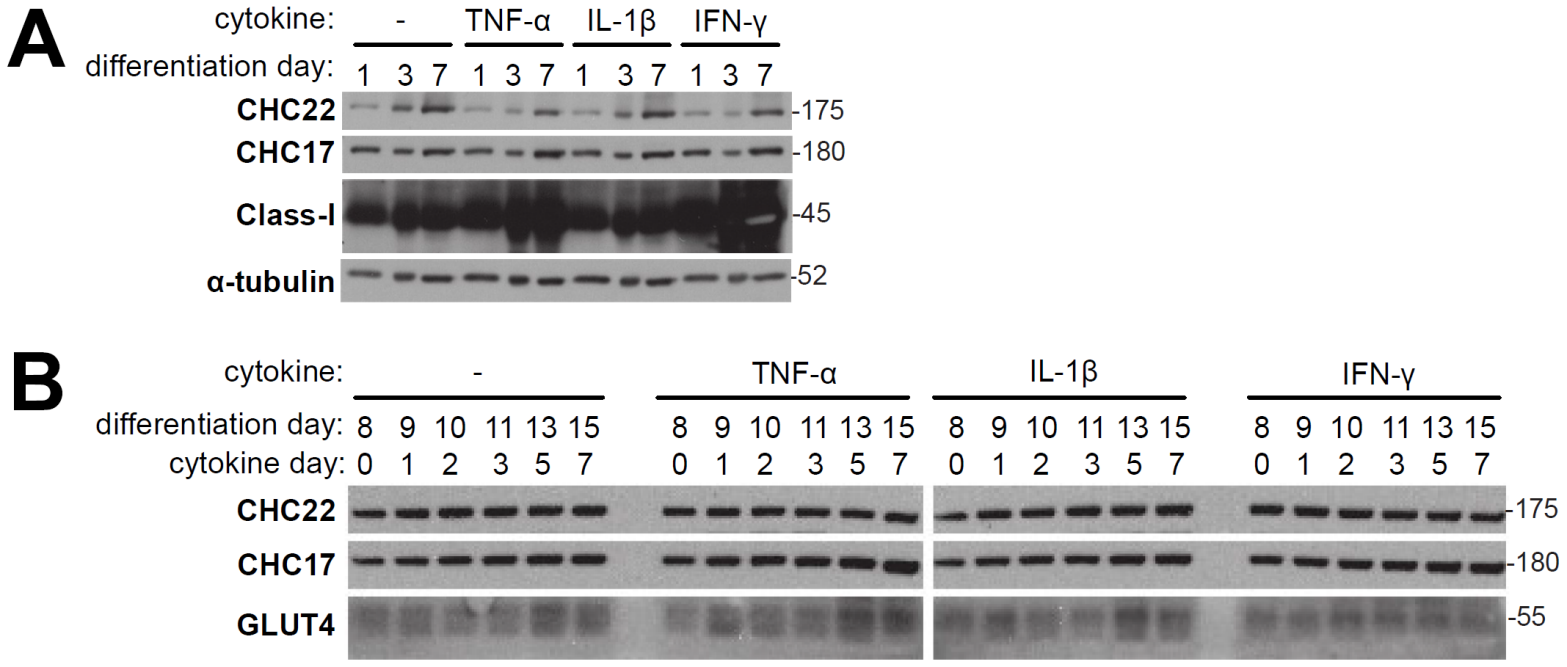
Effect of cytokines on human myoblast or myotube cultures. A) LHCNM2 human skeletal muscle myoblasts were cultured under differentiation conditions for the indicated number of days in the presence or absence (−) of the indicated cytokines. Cell lysates prepared on the indicated day were analyzed by immunoblotting for the protein indicated at the left. B) Experiment as in A with addition of indicated cytokines on differentiation day 8 when myotubes had formed. Molecular mass (kilodaltons) of the proteins detected is indicated at the right.

LHCNM2 human skeletal muscle cells were a gift from W. Wright, University of Texas Southwestern Medical Center [1], [14], [24]. Cells were grown on rat-tail collagen coated flasks in basal media (4∶1 DMEM:M199, 20 mM M HEPES, 0.03 mg/l ZnSO_4_, 1.4 mg/l Vitamin B12) supplemented with 10% FBS, 55 mg/l dexamethasone, 2.5 ng/ml HGF, 50 µg/ml penicillin, 50 µg/ml streptomycin and 0.625 mg/l fungizone (growth media). Differentiation was achieved by switching media to early differentiation media on day 0 (basal media supplemented with 0.5% FBS, 10 mg/l insulin, 50 mg/l apo-transferrin and 5.5 mg/l dexamethasone) and later differentiation media (basal media supplemented with 0.5% FBS, 10 mg/l insulin, 50 mg/l apo-transferrin and 55 mg/l dexamethasone). Later differentiation media was used as soon as multinucleated myotubes appeared in the culture, normally after about 7 days. Cells were differentiated for an additional 7 days. TNF-α (10 ng/ml), IL-1β (50 pg/ml) and IFN-γ (50 ng/ml) were included in the cultures as described in the text and the legend to Figure 4.

### NADH-tetrazolium Reductase (NADH-TR) Staining of Mouse Muscle Sections

Transverse sections from snap-frozen muscle samples, day 28 after injury, were stained for myofiber type [22]. Fibers were identified as red, intermediate or white by setting fixed parameters for all the sections and cross-sectional area was determined for each fiber, using ImageJ. Tissue sections were scored blind and sections from three CHC22-mice or three WT mice were scored, representing an approximate total of 1500 fibers from each mouse type.

### Mouse Primary Myoblast Culture

Primary myoblasts were derived from the hind limb muscles of WT and CHC22-transgenic mice [23]. To induce differentiation and fusion, myoblasts were seeded at a density of 2×10^5^ cells/well on dishes coated with entactin, collagen IV and laminin (E-C-L; Upstate Biotechnology) in growth media [GM: DME, 10% FBS, 10 mM HEPES, 50 µg/ml penicillin, and 50 µg/ml streptomycin], then switched to fusion media [FM: DME, 1% insulin-transferrin-selenium-A supplement (Invitrogen)], 100 U/ml penicillin G and 100 µg/ml streptomycin) with low (5.6 mM) or high (25 mM) glucose. At indicated time points after the switch to FM, cells were immunostained by peroxidase using antibody against eMHC (F1.652; Developmental Studies Hybridoma Bank) and analyzed as described [24]. At least 800 nuclei were analyzed for each culture condition and myoblasts from three independent isolations from both CHC22 and WT mice were assayed.

### Statistical Analysis

Statistical significance between two groups or among multiple groups was evaluated using Student’s t test, Fisher’s exact test, Tukey-Kramer’s test after the F-test, or one-way or two-way analysis of variance (ANOVA) by Microsoft Excel, Graphpad, Prism or js-STAR2012 (ver.2.00j). For each figure, the test used is specified in the legend.

## Results

### CHC22 and Components of the GLUT4 Compartment are Increased and Co-localized in Regenerating Human Muscle

Detection of elevated levels of CHC22 in recovering injured rat muscle suggested that pathways involving CHC22 could be involved in regeneration [7]. To explore this possibility for human tissue, CHC22 expression was analyzed in skeletal muscle sections from patients with several different myopathies. The idiopathic inflammatory myopathies polymyositis (PM, *n = *4) and dermatomyositis (DM, *n = *3) were chosen for analysis because they are characterized by muscle tissue with high levels of inflammation and an active regeneration response [19]. Sections from patients with limb girdle muscular dystrophy (LGMD, *n* = 3) were also examined to study a non-inflammatory muscle myopathy. LGMDs are autosomal muscular dystrophies encompassing a large number of rare disorders with a common phenotype of adult-onset with slowly progressive weakness involving shoulder or pelvic-girdle muscles [25]. The LGMD patients in our study, although not having been diagnosed with specific genetic defects, had a family history of affected siblings and were positive for dystrophin immunostaining, distinguishing them from the more common form of muscular dystrophy, X-linked Duchenne’s muscular dystrophy. In patients’ tissue, regenerating myofibers were identified by immunostaining for embryonic myosin heavy chain (eMHC) and central nuclei [26], commonly used markers for skeletal muscle regeneration (Figure 1). Compared to mature eMHC-negative myofibers, regenerating eMHC-positive myofibers in sections of all three myopathies displayed more intense CHC22 immunostaining. This CHC22 staining was primarily in intracellular structures and localized in the interior of the fiber (Figures 1A–F). In contrast, CHC22 immunostaining in mature fibers and in the control sample was mainly concentrated at the plasma membrane. For the three patient tissues shown in Figures 1B–D, quantitative analysis of fiber staining (in several fields) revealed that eMHC-positive fibers had significantly more intense total CHC22 staining and increased internal staining (Figures 1E and 1F), compared to eMHC-negative fibers from the same patients. When muscle samples from three DM patients and four PM patients were analyzed for double labeling of eMHC and CHC22, the frequency of intense internal CHC22 immunostaining was significantly higher in eMHC-positive muscle fibers than in eMHC-negative myofibers (about 65–70% vs 10–20%, Figure 1G).

The role of CHC22 in GLUT4 trafficking pathways in human myoblasts and adipocytes prompted a comparison of CHC22 and GLUT4 expression in regenerating human skeletal muscle. In sections from patients with PM, regenerating myofibers that were labeled for eMHC also displayed intense GLUT4 immunostaining with an interior distribution similar to that of CHC22 (Figure 2A, magnified box) and the two proteins were partially co-localized (Figure 2B, magnified box). Quantification of additional myofibers from the sections shown in Figure 2A and 2B confirmed increased internal GLUT4 in fibers that were eMHC-positive or positive for internal CHC22. Ubiquitous CHC17 clathrin was not more prominent in myofibers with high levels of GLUT4 compared to other fibers throughout the muscle sections examined and, although quantification suggested some increase along with GLUT4, this was not statistically significant (Figure 2C, magnified box). The differential expression pattern of CHC22 and CHC17 is consistent with the clear delineation of function of these two clathrins in human skeletal muscle [1], [14]. The observation that both CHC22 and GLUT4 staining is shifted internally in regenerating myofibers is consistent with the established perinuclear localization of GLUT4-containing intracellular compartments, since nuclei in regenerating muscle are more centrally located, while mature fibers are characterized by sub-sarcolemmal nuclei (26).

Following up the implication of an amplified GLUT4 transport pathway in regenerating fibers, sections from the same patients were immunolabeled to localize VAMP2. In small regenerating myofibers identified by central nuclei [26] and high levels of intracellular CHC22, VAMP2 immunostaining was intense throughout the cytoplasm and co-localized extensively with CHC22 (Figure 2D, magnified box). In larger regenerating myofibers with elevated levels of intracellular CHC22, VAMP2 co-localization with CHC22 was also clearly observed (Figure 2E, magnified box). Both proteins displayed punctate immunostaining (Figures 2D and 2E, magnified boxes) and quantification confirmed a significant increase in internal VAMP2 in fibers that were positive for internal CHC22. Enhanced expression of VAMP2 has been described for regenerating rat myofibers [11], and the observations here demonstrate that this is also a characteristic of human regenerating myofibers. Thus three components of the GLUT4 trafficking machinery, CHC22, GLUT4 and VAMP2, were all elevated in the interior of human regenerating myofibers.

The presence of CHC22 and GLUT4 in regenerating fibers defined by expression of eMHC and central nuclei raised the question of whether satellite cells participating in regeneration also express CHC22 and GLUT4. We therefore characterized distribution of these proteins compared to the satellite cell marker Pax7 [27], analyzing muscle tissue from two more sets of patients, comprising a new cohort of DM patients (*n* = 3), as well as patients with necrotizing myopathy (NM, *n* = 3). NM is characterized by the presence of necrotic and regenerating fibers in the absence of significant inflammation. For Pax7 expression analysis, the DM and NM patient samples were selected for a high content of regenerating fibers, visible upon H & E staining, and we confirmed that these samples had abundant fibers that co-stained for intracellular CHC22 and GLUT4 (Figure S1). The selected samples were then triple-labeled for Pax7, CHC22 and GLUT4 (Figure 3). Pax7-positive cells were observed associated with the rims of myofibers in both disease states (Figure 3A). In DM, almost 100% of Pax7-positive cells displayed strong labeling for both CHC22 and GLUT4 and for NM >80% of Pax7-positive cells showed intense labeling for all three markers (Figure 3A arrowheads and Figure 3B). Together these human tissue studies suggest a link between CHC22 and its associated GLUT4 pathway in the muscle regeneration process. They further suggest that CHC22 could be a reliable marker for regenerating fibers in human muscle.

### Inflammatory Cytokines Present in Damaged Muscle do not Affect CHC22 Expression

Tumor necrosis factor (TNF)-α, a multi-functional cytokine, is elevated in muscle of patients with PM, DM and Duchenne’s muscular dystrophy, and expression of TNF-α correlates with levels of muscle regeneration [28]. TNF-α signaling through NF-κB and p38MAPK has been shown to increase the translocation of GLUT4 vesicles to the plasma membrane in rat L6 muscle cells [29]. Given this connection between TNF-α, muscle regeneration and glucose metabolism, we investigated whether expression of TNF-α might be responsible for the elevated levels of CHC22 and GLUT4 in regenerating myofibers. To address this, we added TNF-α to differentiating human myoblast cultures and assessed expression levels of CHC22 and GLUT4. For comparison, the effects of other inflammatory cytokines, interleukin 1β (IL-1β) and interferon gamma (IFN-γ), were also analyzed. IL-1β is also increased in muscle of patients with inflammatory myopathies, while levels of IFN-γ are not particularly elevated [19]. Exogenous cytokines were added to human myoblasts (LHCNM2 cells) [1], [14], [30] either on day 1 of switching to differentiation media or on day 9 of differentiation and cells cultured further, collecting samples for analysis on progressive days of differentiation (Figure 4). As we previously observed, CHC22 levels increased during days 1–9 of differentiation [15]. TNF-α and IFN-γ had the expected effect of strongly increasing expression of class I histocompatibility molecules (Class-I), evaluated as a positive control (Figure 4A) [31]–[33]. However, levels of CHC22, CHC17 and GLUT4 expressed in the presence of each cytokine were comparable to control levels of expression at each time point (Figure 4). These culture experiments suggest the dramatic increase observed for CHC22 and GLUT4 immunofluorescence in regenerating fibers is not a response to inflammatory cytokines, consistent with the fact that increase of both markers was also observed in the non-inflammatory human myopathies (LGMD and NM). We hypothesize that these observed increases reflect a role for upregulation of the GLUT4 pathway that is intrinsic to muscle regeneration.

### Levels of GLUT4 Pathway Components Increase in Regenerating Muscle from Wild-type and Transgenic Mice

**Figure 5:**
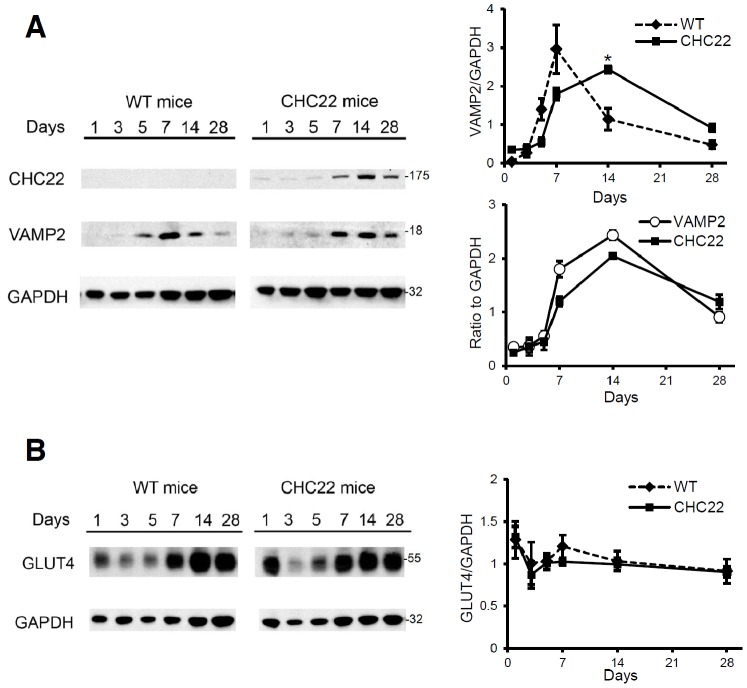
Elevation of components of the GLUT4 trafficking pathway during mouse skeletal muscle regeneration. A) VAMP2 and CHC22 levels during muscle regeneration after cardiotoxin injection on day 0 were compared between WT and CHC22-mice in muscle samples harvested on the day indicated. One typical set of immunoblots is shown at the left and quantification of VAMP2 relative to GAPDH signals from three experiments generated the ratios plotted at the right (upper plot). There was a statistically significant difference between WT and CHC22-mice in VAMP2 expression on day 14 after cardiotoxin injection (*p<0.05), as determined by Student’s t test. Quantification of VAMP2 and CHC22 relative to GAPDH in the CHC22-mice shown at the left was plotted (right, bottom). B) GLUT4 levels during muscle regeneration for wild type and CHC22-mice (n = 3). One typical set of immunoblots is shown at the left and quantification of GLUT4 relative to GAPDH signals is plotted on the right. No significant difference between WT and CHC22-mice was detected. Molecular mass (kilodaltons) of the proteins detected is indicated at the right.

The gene encoding CHC22 is a pseudogene in mice, a property shared by sixteen strains of wild mice, as well as laboratory mice [16]. In mouse muscle, the GSC is formed in the absence of CHC22, relying on CHC17-mediated transport [17], [34]. Transgenic mice that express CHC22 under the control of its endogenous human promoter have been produced and characterized (CHC22-mice). In these mice, CHC22 protein was detected in muscle and adipose tissue, and not in other tissues by immunoblotting [1]. Upon further histological analysis (data not shown), CHC22 was also detected in a subset of cerebellar neurons but not motor neurons, consistent with the presence of the GLUT4 pathway in some cerebellar neurons [35]. We previously showed that, in CHC22-mice, the presence of CHC22 in muscle traps GLUT4 in an intracellular compartment, resulting in less GLUT4 on the muscle cell surface under ad lib feeding conditions [1]. To determine whether CHC22 expression in the transgenic mice parallels that observed for human regenerating muscle fibers, muscle degeneration and regeneration were induced by injection of cardiotoxin into the *tibialis anterior* (TA) muscle. This insult causes extensive and reproducible muscle necrotic injury and the regeneration program that ensues is well documented [36]. After cardiotoxin injection, satellite cell proliferation occurs within two days, myogenic differentiation is initiated within three days, new myofiber formation is evident within five days, and muscle architecture is largely restored within ten days [36]. In cardiotoxin-treated TA muscle from CHC22-mice, expression of CHC22 increased upon muscle regeneration, as assessed by quantification of immunoblotting experiments (Figure 5A). Thus factors regulating CHC22 expression in regenerating human muscle are also present in regenerating mouse muscle and can stimulate the human CHC22 promoter.

To determine whether other components of the GLUT4 pathway were increased during mouse muscle regeneration, the expression levels of VAMP2 (Figure 5A) and GLUT4 (Figure 5B) were also analyzed. In WT mice, VAMP2 levels increased dramatically, starting on day 5 after injection, peaking at day 7, and decreasing afterwards. In regenerating muscle of CHC22-mice, there was an apparent delay in the increase of VAMP2 expression, and elevated VAMP2 persisted longer during the regeneration period compared to WT mice (Figure 5A). Our previous studies indicated increased stability of VAMP2 in untreated muscle of CHC22-mice [1]. This might explain the observed change in VAMP2 behavior in the presence of CHC22 because the changes in VAMP2 level correlated with changes in CHC22 level (Figure 5A). This observation is consistent with the expanded and partially defective GSC in the CHC22-mice that we described previously [1]. For the CHC22-mice, increased expression of GLUT4 was observed during muscle regeneration with no significant differences from the expression pattern seen for WT mice (Figure 5B). For both WT and CHC22-mice, GLUT4 levels were down on days 3 and 5, after toxin injection caused necrosis. GLUT4 levels then increased as regeneration proceeded, peaking on day 14, but remaining at levels higher than those observed one day post-injury, even at day 28, unlike the dramatic drop in VAMP2. The increase in components of the GLUT4 pathway in wild-type and CHC22-mice parallels that observed for rat [7] and human (Figures 1–3) regenerating muscle. Notably, in the CHC22-mice, CHC22 expression behaves as a component of the GLUT4 pathway. However, because of the CHC22 expression, GLUT4 transport is impaired in their skeletal muscle [1], so the CHC22-mice can be analyzed to assess whether GLUT4 traffic contributes to muscle regeneration.

### Delayed Maturation of Regenerating Fibers in Mice Expressing CHC22

**Figure 6:**
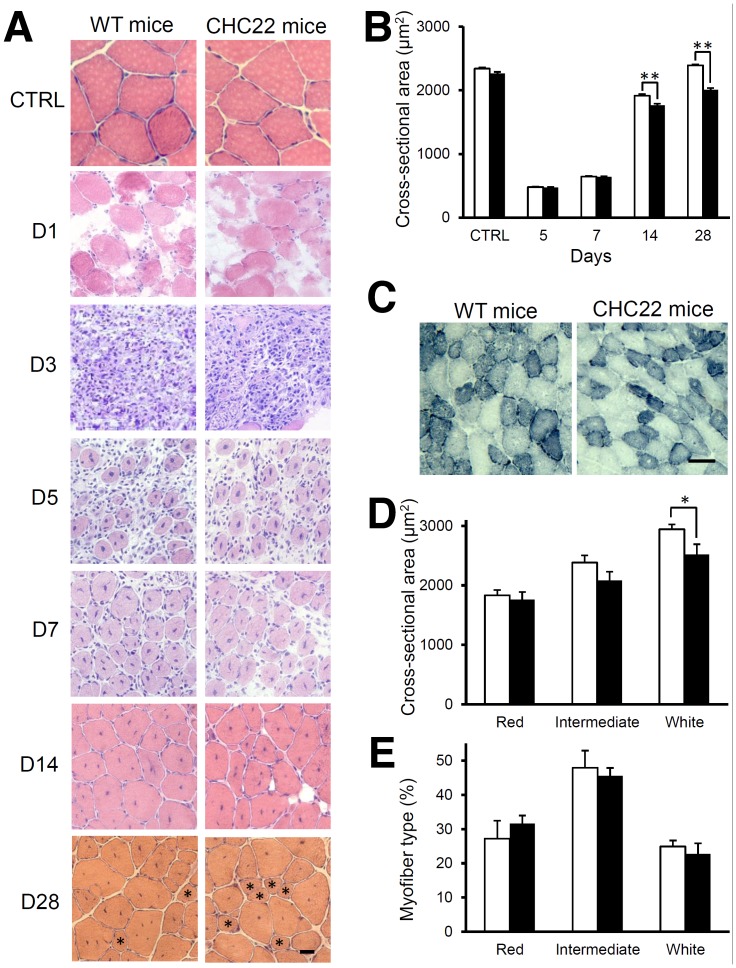
Delayed maturation and fiber type analysis of regenerating fibers in CHC22-transgenic mice. A) Hematoxylin and eosin staining of transverse muscle sections from WT or CHC22 transgenic mice after cardiotoxin injection at day 0, on the indicated days (D). Control (CTRL) sections were prepared from uninjured muscle. On day 28, some CHC22 myofibers were similar in cross-sectional area and diameter to WT myofibers; however, smaller myofibers (<40 µm diameter, marked by asterisks) were more frequently observed in the CHC22-mice compared to WT mice (scale bar, 20 µm; *n = *3). B) The mean cross-sectional area of myofibers with centrally located nuclei from WT (white bars) and CHC22-mice (black bars) was calculated and plotted for each indicated day after cardiotoxin injection. Control mice for each strain were not injected with cardiotoxin. There was a statistically significant difference in the average fiber cross-sectional area between WT mice and CHC22-mice on days 14 and 28 after injection (*n* = 3, evaluating 1200–2000 myofibers per mouse; **p<0.01), as determined by Student’s t test. C) Transverse sections of muscle from CHC22-mice and WT mice, harvested 28 days after cardiotoxin injection, were stained for NADH-TR in order to determine the myofiber type. Oxidative (red) myofibers appear dark, glycolytic (white) myofibers appear light and intermediate (pink) myofibers appear intermediate in color. D) Fibers in 28-day regenerating muscle from CHC22-mice (black bars) and WT mice (white bars) were classified by type and their cross-sectional area measured in pixels using ImageJ (n = 3, evaluating ∼1500 fibers per mouse, *p<0.05, by Student’s t test). E) The percent of each fiber type in 28-day regenerating muscle from CHC22-mice (black bars) and WT mice (white bars) from the analysis in D is plotted.

To determine whether muscle regeneration is affected in the CHC22-mice, the cross-sectional area and the diameter of regenerated myofibers, were analyzed over a time course following injury by cardiotoxin, as it is documented that these parameters increase progressively as regeneration occurs. Regenerating myofibers were also identified by centrally located nuclei [26]. Based on this analysis, CHC22 mice exhibited delayed late-stage muscle regeneration compared to WT mice (Table 1, Fig. 6). On days 5 and 7, regenerating muscle fibers of WT mice and CHC22 mice were comparable in size as shown in H&E staining (Figure 6A) and in their average cross-sectional area (Figure 6B) or diameter (Table 1). However, on days 14 and 28 after cardiotoxin injection, CHC22 mice had significantly smaller regenerated muscle fibers in contrast to WT mice (Figure 6B, Table 1). Especially on day 28, small regenerated muscle fibers with central nuclei were more frequently observed in CHC22 mice compared to WT mice (Figure 6A, shown by asterisks). The GLUT4 response in the CHC22-mice is diminished but not completely impaired [1]. This correlates with the modest degree of reduction in muscle regeneration at late stages of recovery from toxin injection, but still suggests that fiber maturation is affected by disruption of GLUT4 traffic in these mice.

**Table 1:** Mean diameter (microns) of regenerating muscle fibers in wild type mice or CHC22 transgenic mice on indicated days after cardiotoxin injection.

		Control#	Day 5	Day 7	Day 14	Day 28
WTmice	mean ±s.e.m.	71.1±0.5	31.2±0.2	38.0±0.2	53.0±0.5	71.0±0.4
	n^+^	1268	1164	1642	1799	2012
CHC22mice	mean ±s.e.m.	69.4±0.4	30.4±0.1	37.1±0.2	47.0±0.3**	63.6±0.4**
	n	1308	1125	1669	1800	1988

# Control animals were not injected with cardiotoxin.

n^+^ = total number of muscle fibers analyzed from 3 animals each of WT or CHC22-mice.

** *P*<0.01, by Student’s t test.

The presence of some smaller myofibers and some normal myofibers in the CHC22 regenerating muscle, suggests that regeneration of a subpopulation of myofibers is affected following injury. To address this hypothesis, the fiber type composition of muscle removed 28 days after injury was determined for WT and CHC22-mice and the cross-sectional areas for each fiber type were measured. Muscle sections were stained for NADH-TR, an indicator of mitochondrial activity. From this procedure, oxidative (red) myofibers stain darkly and glycolytic (white) myofibers stain lightly, and intermediate fibers (pink) show intermediate coloration (Figure 6C). In injured muscle of CHC22 mice (12 weeks of age), the glycolytic fibers had a significantly smaller cross-sectional area (Figure 6D), though fiber type composition was unchanged (Figure 6E).

### Myoblasts from CHC22-mice Fuse but do not Proliferate in Response to Glucose

**Figure 7:**
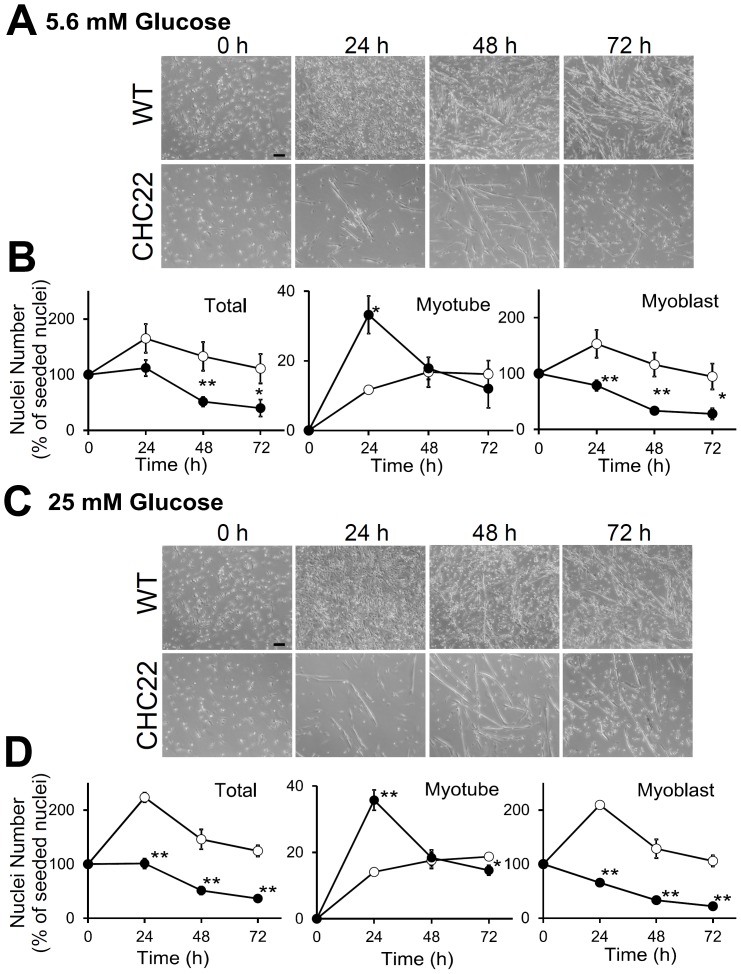
Myoblasts from CHC22-mice undergo fusion but do not exhibit glucose-dependent proliferation. A and C) Images of primary myoblasts from wild-type (WT) or CHC22-transgenic mice cultured in FM with A) low (5.6 mM) or C) high (25 mM) glucose for the indicated time in hours (h), all seeded at the same density (scale bars, 100 µm). B and D) At the indicated time period for myoblasts cultured as in A and C, the nuclei were quantified for total number (Total), number present in multi-nuclear myotubes (Myotube), and number in mono-nuclear cells (Myoblast). These quantifications are plotted relative to the total nuclei present at the start of differentiation (switch to FM at 0 hours) for cultured myoblasts from CHC22-mice (filled circles) and WT mice (open circles). Two-way ANOVA and Tukey-Kramer post-hoc test showed significant differences between WT and CHC22 in all three panels. (*p<0.05 and **p<0.01) at indicated time points.

The delayed regeneration of glycolytic fibers in CHC22 mice might be a direct effect of their impaired GLUT4-mediated glucose uptake pathway. Oxidative fibers express more GLUT4 [38]–[40], so these fibers would potentially be less affected by the over-sequestration of GLUT4 induced by the presence of CHC22. To investigate whether the delay in regeneration observed in CHC22 mice could be explained by GLUT4 pathway defects, myoblasts isolated from CHC22 mice were compared to WT myoblasts during growth in fusion media (FM), in which they form differentiated myotubes (Figure 7). Isolated myoblasts from CHC22− or WT mice were cultured in FM with either low (5.6 mM) or high glucose (25 mM). After 24 h culture in either glucose concentration, myoblasts from both CHC22-mice or WT mice had started to fuse into small nascent myotubes with two to three nuclei. By 48 h, larger mature myotubes with many nuclei had formed in both CHC22 and WT cultures (Figure 7A and C). After 24 h of culture in FM at either glucose concentration, about 35% of the CHC22 myonuclei were present in myotubes, suggesting that the mechanics of fusion function properly in CHC22-mouse muscle (Figure 7B and D). In fact, compared to the WT myoblasts, fusion of CHC22 myoblasts was increased under either glucose condition, with a larger percentage of nuclei present in myotubes. Enhanced fusion rate is a hallmark of myoblasts under stress [41]. However, in contrast to the CHC22 myoblasts and myotubes, the WT myoblasts continued to proliferate in culture from 0–24 h, so that their overall numbers increased, while the CHC22 myoblasts remained stable for 24 h and then their numbers started to decline (Figure 7B and D). After 72 h of culture, damaged myotubes were visible in the cultures from CHC22-mice but were absent in cultures from WT mice (arrowheads, Figure S2). WT myoblasts showed a greater increase in numbers after 24 hours (total nuclei) when cultured in high glucose (> twofold) compared to low glucose (< twofold) (Figure 7B vs D). This increased proliferation in response to increased glucose was not observed for the CHC22 myoblasts, suggesting that impaired glucose uptake affects their proliferation, and could account for delayed regeneration.

### Fiber Type Switch during Aging of CHC22-mice

**Figure 8:**
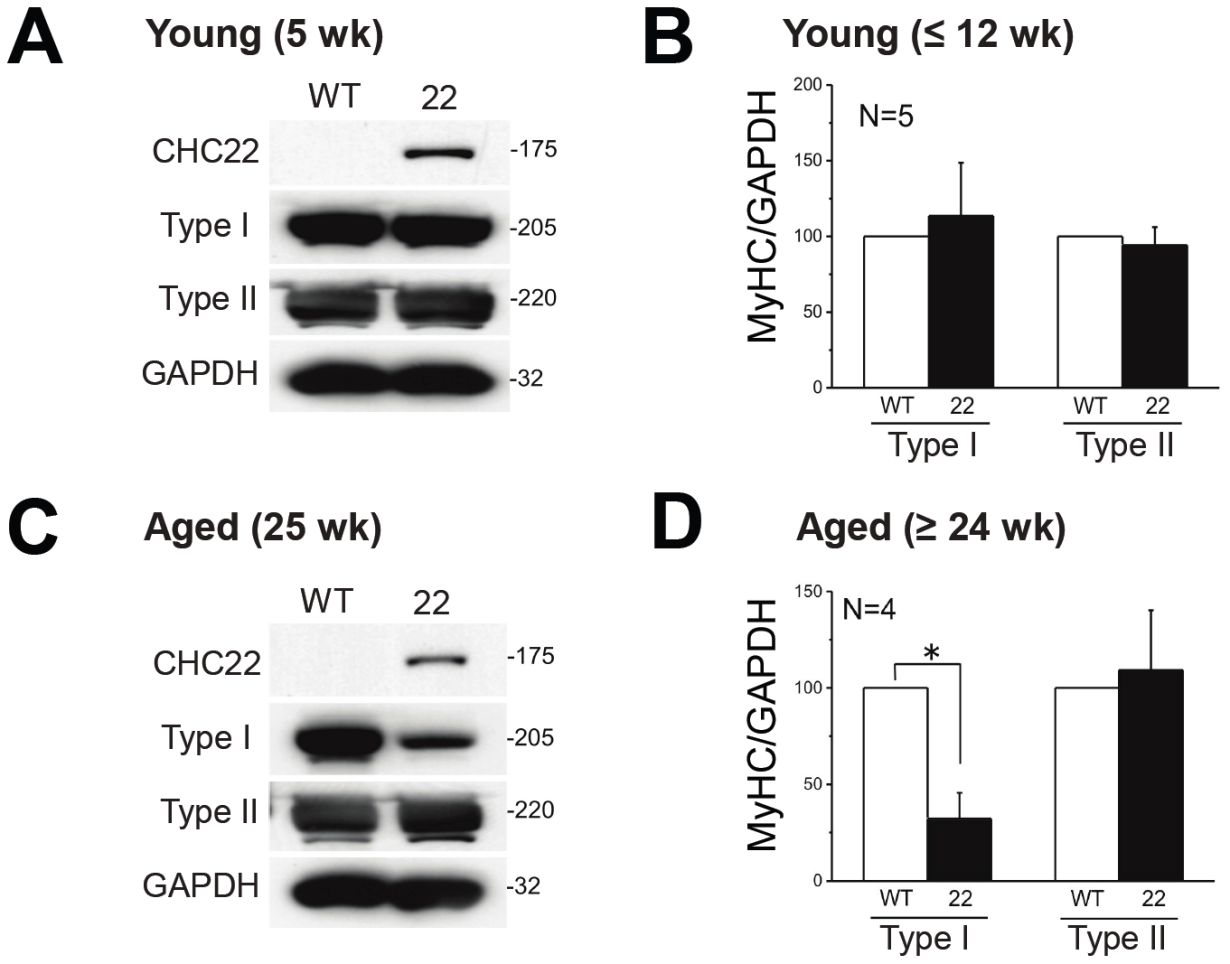
Muscle fiber type switch during aging of CHC22-mice. Skeletal muscle (*gastrocnemius*) was harvested from CHC22-mice and WT mice at the indicated ages and tissue homogenate was analyzed by immunoblotting for Type I and Type IIa/b myosin heavy chains, as well as for CHC22 and loading control GAPDH. A typical immunoblot for each age group is shown at the left. Signals for myosin heavy chain (MyHC) type levels relative to GAPDH signals are shown at the right for 4–5 animals from the indicated age groups. The drop in Type I myosin heavy chain in aged CHC22 mice is significant, *p<0.05, by Student’s t test.

We observed fiber type differences in response to muscle injury in CHC22-mice (Figure 6), suggesting that their poor myotube survival and myoblast insensitivity to glucose (Figure 7) might affect myofiber types differently. To address this, we examined the fiber type composition of muscle from uninjured CHC22 mice over time, using myosin isoforms as fiber type markers (Figure 8). Oxidative fibers express type I myosin, while intermediate and glycolytic fibers express type IIa and IIb myosins, respectively [37]. CHC22 mice that were twelve weeks or younger had myosin expression similar to WT mice. However, aged CHC22-mice (>24 weeks) had a decreased level of Type I myosin compared to age-matched WT mice (Figure 8). A switch from oxidative to glycolytic muscle is reported to occur in patients with type 2 diabetes [38], so this elevated glycolytic muscle phenotype for aged CHC22-mice is consistent with their development of symptoms of type 2 diabetes (hyperglycemia and sequestration of intracellular GLUT4) by 20 weeks of age [1]. In younger CHC22-mice (12 weeks), the glycolytic fibers recovered from injury more slowly than glycolytic fibers in WT mice of the same age (Figure 6), while oxidative fibers showed no difference in response to injury. With age, however, the survival of the oxidative fibers was decreased (Figure 8). These phenotypes suggest that the impaired GLUT4 pathway of the CHC22-myoblasts leads to decreased growth rate of the more glucose-dependent glycolytic fibers but also decreases the long-term survival of the more robust oxidative fibers, which are more insulin-dependent.

## Discussion

Muscle growth and maturation depend on numerous factors, including uptake of glucose through the glucose transporter GLUT4 [42]. A role for GLUT4-mediated glucose uptake during muscle regeneration has also been proposed [8]. Here we analyze components of the GLUT4 membrane traffic pathway and their expression in regenerating human muscle, as well as assess a role for the GLUT4 pathway in muscle regeneration in a transgenic mouse model. We present evidence that membrane traffic of GLUT4 is increasingly active during human and mouse muscle regeneration and show that in CHC22-transgenic mice with impaired GLUT4 membrane traffic in their skeletal muscle, regeneration is delayed. The myoblasts from CHC22-mice do not increase their proliferation in response to increased glucose, which promotes proliferation of myoblasts from WT mice, suggesting a connection between the impaired GLUT4 pathway in the CHC22-transgenic animals and their impaired muscle regeneration. The skeletal muscle of aged CHC22-mice also had an increased percentage of glycolytic fibers, as seen in some patients with type 2 diabetes [38]. Together these findings support a role for GLUT4 function during muscle regeneration in humans and mice and in maintenance of fiber type, at least in mice. In addition, we show that markers of the GLUT4 pathway, including GLUT4, CHC22 and VAMP2 are diagnostic for regenerating human myofibers.

The GLUT4 transporter is sequestered in a GSC until it is released in response to insulin stimulation, so its membrane traffic is highly specialized. In humans, GSC formation in skeletal muscle and adipocytes involves the CHC22 isoform of clathrin that is missing from mice, defining a species-restricted aspect to GLUT4 membrane traffic [1]. Following up the demonstration that CHC22 clathrin is a necessary component of the human GLUT4 pathway, we investigated whether the increased CHC22 levels, previously observed in regenerating rat muscle [7], indicated upregulation of the GLUT4 pathway during regeneration. Analysis of regenerating muscle fibers in patients with four different human myopathies revealed that levels of CHC22, GLUT4 and VAMP2 are elevated during regeneration. These proteins also have an altered internal distribution in regenerating fibers compared to mature fibers in the same tissue, which can be explained by repositioning of the nuclei and associated perinuclear regions in regenerating muscle [26]. The elevation of GLUT4 pathway markers, combined with more visible staining of the GSC region, suggests that active membrane traffic of GLUT4 and regeneration of the GSC is occurring during human muscle regeneration. High levels of CHC22 and GLUT4 in Pax7-positive cells suggested that this pathway is amplified early in the regenerative process.

The mechanism by which the GLUT4 pathway components are stimulated is not clear. Earlier studies of rat muscle regeneration revealed an interaction between the regulatory GLUT4 enhancer sequence and transcription factors that are induced during muscle regeneration [8], but the regulatory sequences that affect CHC22 expression are not known. Here we investigated whether signaling via inflammatory cytokines that are present in regenerating muscle might influence expression of CHC22 and we observed no effect of TNF-α, IL-1β or IFN-γ on expression of CHC22 in cultured human myotubes. Although TNF-α was reported to influence GLUT4 expression in rat muscle [29], no obvious effect was observed in cultured human myoblasts and myotubes, nor did other cytokines affect GLUT4 expression in these cultures. Thus, we suggest that inflammation is not responsible for the observed increase in GLUT4 transport components in regenerating human muscle and propose that the increase results from some muscle-intrinsic program during regeneration. The time course of GLUT4 pathway expansion in regenerating mouse muscle coincides with the time course for innervation (data not shown), suggesting that the pathway might expand to meet the increased energy needs that accompany contraction.

We have previously demonstrated that transgenic mice expressing CHC22 in their skeletal muscle and fat, under the control of its human promoter, display clear defects in GLUT4 trafficking. In their skeletal muscle, presence of the CHC22 transgene causes excessive intracellular sequestration of GLUT4 and VAMP2 and a reduction of GLUT4 at the sarcolemma and T-tubules. These changes are accompanied by age-dependent hyperglycemia in the CHC22-mice [1]. With their impairment of GLUT4 traffic and mild diabetic condition, the CHC22-mice qualified as a model system to test the role of the GLUT4 pathway in muscle regeneration. This approach was validated by showing that CHC22 expression is increased upon cardiotoxin injury of skeletal muscle in the CHC22-mice, and that GLUT4 and VAMP2 levels increase in both WT and CHC22-mice after injury, as predicted by our staining of human regenerating muscle. Consistent with previously observed trafficking defects in the GLUT4 pathway of the CHC22-mice [1], the kinetics of VAMP2 expression in their muscle was prolonged after injury compared to injured WT muscle. Importantly, analysis of their skeletal muscle after cardiotoxin injection revealed that the CHC22-mice have a delay in muscle regeneration, characterized by a delay in myofiber maturation. As CHC22, expressed in a tissue-specific fashion from its endogenous human promoter, is not expressed in motor neurons of the transgenic mice (data not shown), the defective regeneration is most likely due to the presence of CHC22 in the muscle fibers themselves and their associated defective GLUT4 traffic. Consistent with this interpretation, we observed that isolated myoblasts from the CHC22-mice were capable of fusing and forming myotubes, but did not proliferate in response to an increase in glucose, as observed for myoblasts from WT mice. Compared to WT myoblasts, the myoblasts from CHC22-mice also had higher fusion rates, typical of myoblasts under stress [41]. We therefore propose that the delayed maturation of CHC22 myofibers during regeneration may be a consequence of the defective GLUT4 pathway in the transgenic mice.

Not all of the regenerating fibers in the CHC22-mice were diminished in size relative to the WT mice, so we compared the fiber types in skeletal muscle of CHC22-mice with WT mice. We found an increase in glycolytic fibers compared to oxidative fibers in aged CHC22-mice, compared to age-matched WT mice, which coincided with onset of hyperglycemia in the former [1]. We also observed that in the injured muscle of CHC22-mice, the glycolytic fibers had decreased cross-sectional area compared to regenerating fibers in WT muscle. Since glycolytic fibers have lower levels of GLUT4 [38]–[40] and are more glucose-dependent, regeneration of the surviving glycolytic fibers could be more sensitive to perturbation of the GLUT4 pathway, accounting for their reduced size compared to regenerated oxidative fibers. Conversely, glycolytic fibers are more reliant on GLUT1 than GLUT4 for glucose import, while oxidative fibers are more dependent on GLUT4 [38]–[40]. Thus over time, oxidative fibers may be less viable due to the impairment of the insulin-responsive GLUT4 pathway that occurs in CHC22 mice, resulting in the alteration of fiber type composition we observed, which mimics that reported for type 2 diabetes. Hyperglycemia is characteristic of aged (>20 weeks old) CHC22-mice [1], so the reduction of oxidative fibers observed in aged CHC22-mice could also be a result of secondary effects of their hyperglycemia. On the other hand, the abnormality in muscle regeneration that we observed following muscle injury of 8-week-old mice is feasibly a direct cause of their impaired GLUT4 pathway rather than due to long term diabetic symptoms. Though we did show defective behavior of myoblasts from CHC22 mice in response to glucose, there may be additional features of the impaired GLUT4 pathway that influence muscle regeneration. For example, the GLUT4 pathway also mobilizes the multi-functional insulin-responsive amino peptidase (IRAP) [44]. Interestingly, the canonical transient receptor potential 3 (TRPC3), a non-selective cation channel, was shown to be involved in insulin-responsive glucose uptake [45] and was also implicated in muscle regeneration [46]. Establishing how the GLUT4 pathway plays a role in muscle regeneration remains a task for the future, but our combined studies of human tissue and a mouse model in which the pathway is defective further support that this pathway contributes to the regenerative process.

## Conclusions

In summary, this work defines a relationship between muscle regeneration and the GLUT4 pathway for glucose metabolism. Increased levels of GLUT4 pathway components are detected in regenerating skeletal muscle fibers of humans and mice, as predicted from earlier studies of rat skeletal muscle. In humans, markers of this pathway include the species-restricted CHC22 isoform of clathrin, which can now be considered a marker of muscle regeneration. Furthermore, we demonstrate a functional role for the GLUT4 pathway in glucose-dependent myoblast proliferation and a negative impact on muscle regeneration when this pathway is impaired. Impairment of the GLUT4 pathway also correlated with long-term changes in muscle fiber type. Together these observations may explain aspects of muscle wasting in patients with insulin-resistant type 2 diabetes [43] and define the GLUT4 pathway as a potential target to stimulate muscle regeneration.

## Supporting Information

Figure S1 **Evidence of regeneration in samples from patients with dermatomyositis and necrotizing myopathy.** (A) Hemotoxylin and eosin (H&E) staining shows numerous regenerating fibers in samples from DM and NM patients, characterized by large nuclei with a non-peripheral location. Examples of regenerating fibers are indicated by black arrowheads. (B) Samples from both patients were double-labeled with antibodies against CHC22 and GLUT4, detected by distinct fluorophores, as in Figure 2A. Regenerating fibers that stain for both markers are indicated by white arrowheads.
(TIF)

Figure S2 **Damaged myotubes in 72 h cultures of myoblasts from CHC22-mice.** Images of primary myoblasts from WT and CHC22-mice cultured 72 h in FM with low (5.6 mM) or high (25 mM) glucose. Regions 1 and 2 are magnified below. White arrowheads indicate cauliflower-like damaged myotubes visible in the cultures from CHC22-mice (scale bars, 100 µm).
(TIF)
