# Supplementary figures and images for: The CHC22 Clathrin-GLUT4 Transport Pathway Contributes to Skeletal Muscle Regeneration
Source: PLoS One. 2013 Oct 30;8(10):e77787. doi: 10.1371/journal.pone.0077787 (PMC3813726; doi:10.1371/journal.pone.0077787)

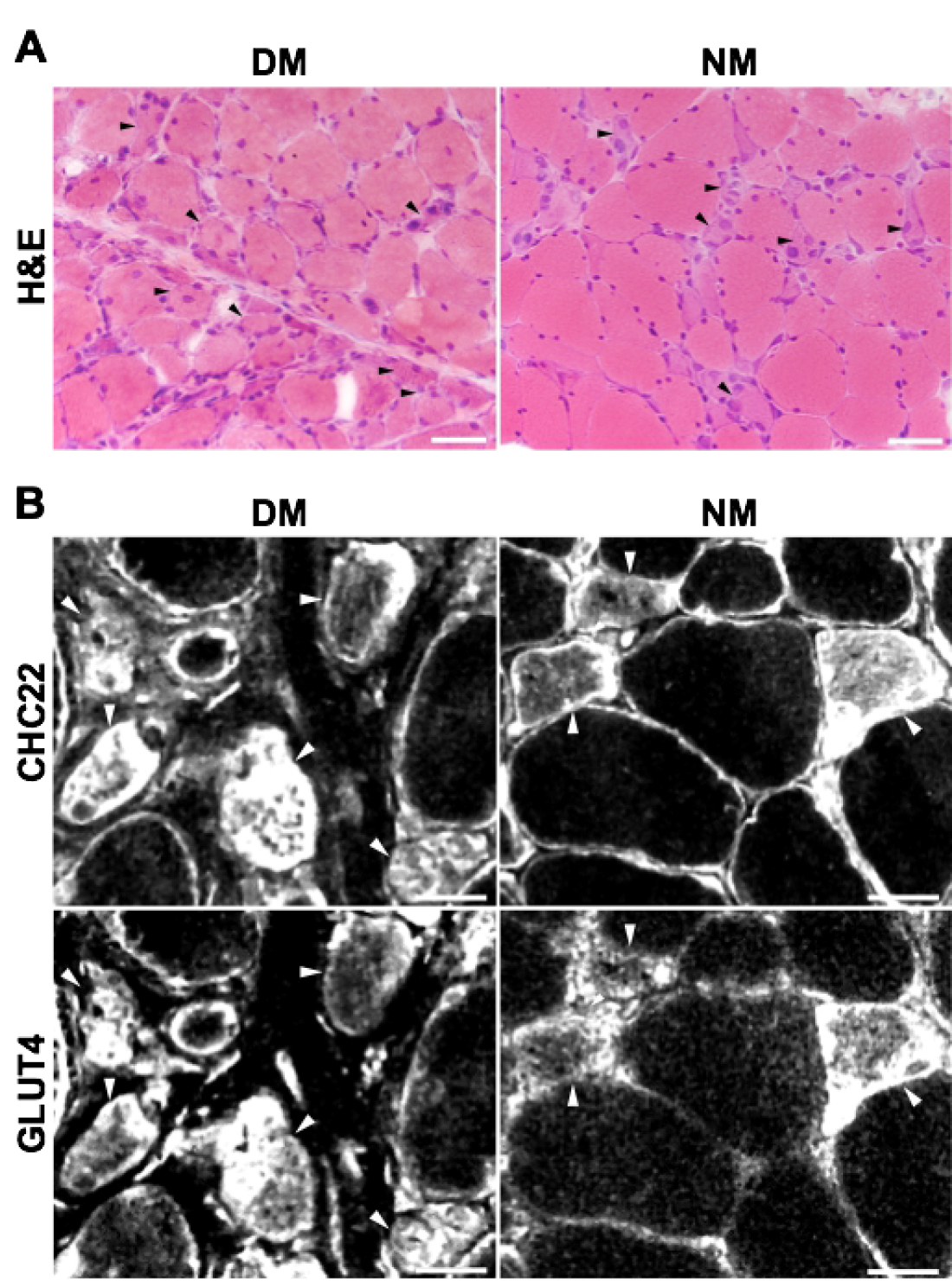

Supplement: Figure S1 — Evidence of regeneration in samples from patients with dermatomyositis and necrotizing myopathy. (A) Hemotoxylin and eosin (H&E) staining shows numerous regenerating fibers in samples from DM and NM patients, characterized by large nuclei with a non-peripheral location. Examples of regenerating fibers are indicated by black arrowheads. (B) Samples from both patients were double-labeled with antibodies against CHC22 and GLUT4, detected by distinct fluorophores, as in Figure 2A. Regenerating fibers that stain for both markers are indicated by white arrowheads. (TIF) [file pone.0077787.s001.tif]

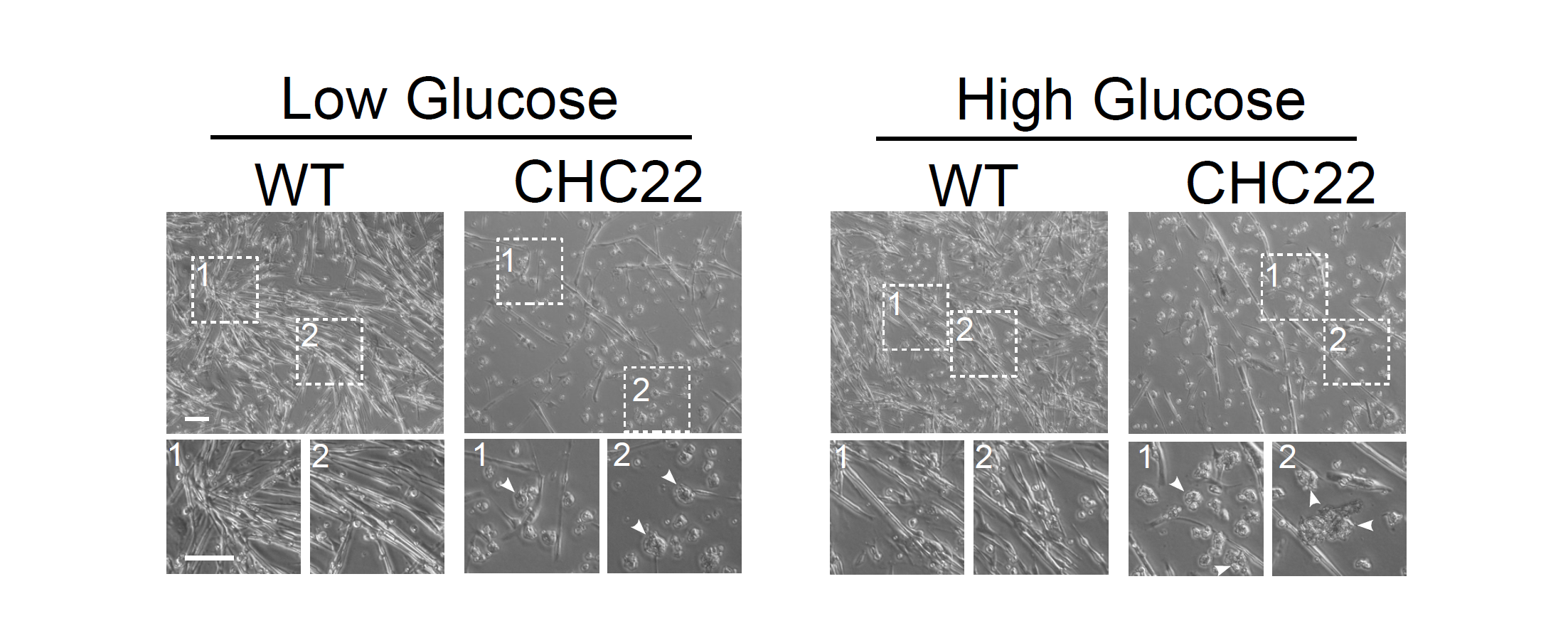

Supplement: Figure S2 — Damaged myotubes in 72 h cultures of myoblasts from CHC22-mice. Images of primary myoblasts from WT and CHC22-mice cultured 72 h in FM with low (5.6 mM) or high (25 mM) glucose. Regions 1 and 2 are magnified below. White arrowheads indicate cauliflower-like damaged myotubes visible in the cultures from CHC22-mice (scale bars, 100 µm). (TIF) [file pone.0077787.s002.tif]
